# Insights into designing educational materials for persons living with dementia: a focus group study

**DOI:** 10.1186/s12877-024-04953-y

**Published:** 2024-04-29

**Authors:** Charity M. Hoffman, Sarah E. Vordenberg, Amanda N. Leggett, Esther Akinyemi, Molly Turnwald, Donovan T. Maust

**Affiliations:** 1 University of Michigan, Ann Arbor, MI USA; 2 Wayne State University, Detroit, MI USA; 3 Ascension Health, Detroit, MI USA

**Keywords:** Benzodiazepines, Deprescribing, Dementia, Alzheimer’s, Cognitive impairment, Polypharmacy, Older adults, Geriatrics, Design, Educational intervention

## Abstract

**Background:**

Persons living with dementia (PLWD) may experience communication difficulties that impact their ability to process written and pictorial information. Patient-facing education may help promote discontinuation of potentially inappropriate medications for older adults without dementia, but it is unclear how to adapt this approach for PLWD. Our objective was to solicit feedback from PLWD and their care partners to gain insights into the design of PLWD-facing deprescribing intervention materials and PLWD-facing education material more broadly.

**Methods:**

We conducted 3 successive focus groups with PLWD aged ≥ 50 (*n* = 12) and their care partners (*n* = 10) between December 2022 and February 2023. Focus groups were recorded and transcripts were analyzed for overarching themes.

**Results:**

We identified 5 key themes: [1] Use images and language consistent with how PLWD perceive themselves; [2] Avoid content that might heighten fear or anxiety; [3] Use straightforward delivery with simple language and images; [4] Direct recipients to additional information; make the next step easy; and [5] Deliver material directly to the PLWD.

**Conclusion:**

PLWD-facing educational material should be addressed directly to PLWD, using plain, non-threatening and accessible language with clean, straightforward formatting.

**Supplementary Information:**

The online version contains supplementary material available at 10.1186/s12877-024-04953-y.

## Background

The number of persons living with dementia (PLWD) in the U.S. is projected to grow to 50 million people by 2050 [1], with 153 million globally by that time [2]. Most healthcare systems are poorly equipped to serve these patients and address their complex medical and psychosocial needs [3], reflected by the fact that PLWD have higher rates of emergency department visits and hospital admissions, with higher treatment costs [4–7].

One factor contributing to PLWD medical complexity is complicated medication regimens that may lack benefit (e.g., cholinesterase inhibitors), lead to harm (e.g., antipsychotics), or pose adherence challenges (e.g., cognitive impairment, difficulty swallowing) [8–11]. The potential for deprescribing—i.e., the intentional de-escalation or discontinuation of a medication in collaboration with a healthcare professional [12]—has been of growing interest to potentially simplify and improve care for PLWD [13]. Psychotropic and opioid medication (“Central nervous system [CNS]-active” hereafter) prescribing to PLWD is of particular concern given the associated increased risks of impaired cognition, falls, respiratory suppression, and even death for older adults. Risks are particularly elevated for PLWD who experience CNS-active polypharmacy (3 or more psychotropic and/or opioid medications) [14, 15], which is common and considered potentially inappropriate for older adults by the American Geriatrics Society Beers Criteria [16, 17].

The EMPOWER study demonstrated that a direct-to-patient educational nudge was an effective intervention to reduce sedative-hypnotic use by older adults without significant cognitive impairment [18]. Educational nudges include carefully framed information designed to influence behavior without removing patient choice [19, 20]. However, existing literature about medication management for PLWD has primarily focused on medication administration by caregivers—as opposed to making decisions about continuing or stopping medications—and interventions have not been co-designed with PLWD or their care partners [21]. Therefore, we sought to adapt the EMPOWER intervention by collaborating with PLWD and their care partners to develop an educational nudge to encourage PLWD who are prescribed CNS-active polypharmacy to talk with their healthcare professional about the appropriateness of their current medication regimen. Our intervention adaptation was guided by FRAME, which is a guide for adapting and modifying interventions [22]. We chose to co-produce the intervention materials with participants, by including PLWD and caregiver perspectives and valuing their knowledge during the design process [23]. This co-production approach was critical because dementia changes cognitive domains including attention, language, and visuospatial abilities (e.g., ability to read and comprehend), which may limit the ability of PLWD to understand complex health-related information and apply it to their own circumstances.

In this article, we discuss lessons learned from a series of focus groups with PLWD and their care partners, which may help inform interventions that include a direct education nudge through written materials. The focus groups were conducted as part of an NIA-funded pilot study of an embedded pragmatic intervention to reduce potentially inappropriate prescribing to PLWD exposed to CNS-active polypharmacy. The overarching goal of the study was to explore whether a direct-to-patient educational nudge co-produced with PLWD and their care partners might increase deprescribing conversations with doctors and ultimately reduce potentially inappropriate medication usage. In this paper, we specifically report on the findings from focus groups conducted with PLWD and their care partners that may help researchers and healthcare systems develop PLWD-facing educational materials.

## Methods

### Study design

This study was developed by a team that included two geriatric psychiatrists, a clinical pharmacist, a social worker, and a gerontologist. We set out to create an informational brochure about the risks associated with CNS-active polypharmacy (i.e., 3 or more psychotropic and/or opioid medications) among PLWD. The initial draft was adapted from the EMPOWER intervention, which addressed sedative-hypnotic prescribing among older adults (without significant cognitive impairment). In addition, while drafting the study brochure, the OPTIMIZE trial [24–26]—which focused on polypharmacy of 5 or more medications from any therapeutic class specifically among PLWD—was published and available as an alternative model. Our goal was to use feedback from three successive focus groups to iteratively modify the draft of the CNS-active polypharmacy brochure. We wanted to ensure that the content was accessible, useful, and motivated recipients (PLWD and their care partners) to initiate a conversation with their doctor about whether all of their prescribed medications continue to be necessary. Consistent with recommendations from the Dementia Engagement and Empowerment Project (DEEP) [27], we enlisted care partners (a supportive family member, partner or friend) to join the PLWD in reviewing materials [28]. In future interventional studies, we plan to deliver written information to PLWD via mail, therefore we provided a copy of the materials via mail ahead of the focus groups.

At least one week prior to the focus group, a copy of the most recent brochure draft was mailed to the participant and his/her care partner. Also included in the mailing was a copy of the consent form and a list of questions to be addressed in the focus group. At the start of the focus group, verbal consent was obtained from each participant. Focus groups took place over video conferencing software (Zoom) and ranged from 60 to 77 min (mean 67 min). Each participant (both the PLWD and care partner) was mailed a $25 check as a thank you for their participation.

### Participants and recruitment

Beginning in November 2022, the study team recruited PLWD and care partners from a variety of sources, including: the University of Michigan Health Research website; the National Council for Dementia Minds (NCDM); or a letter sent to patients who were part of the Michigan Alzheimer’s Disease Research Center (MADRC). Eligibility criteria included: (1) age ≥ 50; (2) ability to read and speak in English; (3) dementia of any type, as reported by the PLWD and/or the care partner (i.e., *Has a doctor ever diagnosed you with dementia?);* and (4) ability and willingness of both PLWD and a care partner to participate in the virtual group discussion. PLWD without a care partner were ineligible to enroll. Recruitment materials informed participants that the research team was seeking to hear the perspectives of PLWD and their care partners as it relates to medications. Information about deprescribing was not included to decrease sampling bias. The research team did not have a pre-existing relationship with participants.

All interested parties completed a brief screening call with a member of the research staff to confirm eligibility; screening calls were completed with the PLWD, their care partner, or both, based on individual comfort.Prospective participants were told that the research team was studying appropriate use of medications in older adults with dementia. During this screening call, participants were asked about their and/or their partner’s gender (PLWD only), age (PLWD only), race/ethnicity and education. One respondent declined to disclose the race of herself and her husband, saying she doesn’t like when people ask about her race. Eligible and interested participants were scheduled for focus groups based on their availability. Focus groups were conducted online to facilitate participation from a geographically dispersed sample. The target number of participants per focus group was 8–12, to include a diversity of perspectives while also ensuring that all participants had an opportunity to speak without feeling overwhelmed or crowded out.

This research was approved by the University of Michigan Institutional Review Board (IRB), which ceded oversight to Advarra, the IRB of Record for the National Institute on Aging’s IMPACT Collaboratory (#Pro00065204).

### Interview guide

All team members collaborated to develop a focus group guide to collect information from PLWD and their care partners (See Additional file 1). The guide focused on a few key areas: (1) content (2), graphical presentation, and (3) physical delivery. Each focus group began with a brief overview of the study and an ice-breaker question (e.g., where they were calling from and a favorite seasonal activity) so participants could virtually meet each other.

For the duration of each focus group, we shared images of the brochure on screen to facilitate discussion of specific content and images. We also encouraged participants to refer to the physical copy of the brochure so they could see the version that would be mailed to future participants. We began by examining the cover together, asking what first caught participants’ attention, and what, if anything resonated. We went on to assess participant reactions to the content itself, including specific language. In addition to asking about the length, we obtained suggestions for content to add or remove. Finally, we asked specific questions about sending the brochure, such as: to whom the material should be addressed (e.g., directly to the PLWD or to them and a care partner) and what might increase the likelihood of our unsolicited mail being read rather than discarded. Finally, we asked PLWD/care partner dyads how decisions about their healthcare get made (e.g., who would be involved in a deprescribing decision?).

### Brochure design

The initial draft of the brochure was designed by the study team, using the EMPOWER and OPTIMIZE brochures as examples. It was a single page, double-sided, 8-in. × 11-in. three panel brochure printed in color on white cardstock. Findings from each successive focus group informed the next iteration of the brochure (i.e., each set of focus group participants reviewed a slightly modified version). Based on cumulative feedback, the final version of the brochure was created by a graphic designer, in consultation with guidance available online about creating materials for older adults, particularly those with cognitive impairment [24, 25]. It was this final version of the brochure that was used on the pilot intervention phase of the research study, which is ongoing (See Additional file 2).

### Data collection & analysis

Focus groups were conducted over a HIPAA-approved video-conferencing platform (Zoom) between December 2022 and February 2023. The first author, a trained qualitative social science researcher, facilitated these focus groups; a second author was also present to observe, ask supplementary questions and take notes when schedules permitted (one co-Investigator each at the first and third focus groups). The facilitators had no prior relationship with focus group participants. To refine the brochure for the three successive rounds, we used notes taken during the focus group to identify initial themes for each of the main categories (i.e., content, graphical presentation, and delivery). Following each focus group, the facilitator immediately typed up hand-written notes, which she clustered into themes and key findings and shared with the full study team. These notes and emerging findings were discussed at team meetings and used to inform additional areas to probe. With participants’ consent, the focus groups were recorded and transcribed verbatim by a transcription software embedded in the recording platform. Employing a rapid qualitative analysis method, the study team coded and analyzed these notes, referring back to specific excerpts from the video recordings to provide context or gain further understanding. Points of disagreement among respondents were further explored in subsequent focus groups. Where there remained disagreement among participants, we weighed the tenor of the feedback, as well as the degree of support each side seemed to elicit from other participants. We adhered to Consolidated Criteria for Reporting Qualitative Research (COREQ) guidelines and included them as relevant [29].

## Results

We conducted three focus groups with a total of 22 participants—12 PLWD and 10 care partners. The average number of participants per focus group was 7.3. While having a care partner willing and able to join the focus group was a condition of participation, 2 partners ended up being unable to participate due to health challenges. Each focus group participant was unique; that is, no single participant participated in more than one focus group. Additional information about participants, based on self-report, is presented in Table 1. At the time of the focus group, two-thirds of the dyads lived in Michigan, while one-third lived elsewhere in the continental U.S.

**Table 1:** Focus group participants

	PLWD (n = 12)	Care Partner (n = 10)
GENDER		
Male	6	*Did not ask*
Female	6	
AGE		
Range	50–85	*Did not ask*
Mean	66.9 years	
RACE		
White	8	8
Black	3	1
Declined to answer	1	1
RELATIONSHIP to PLWD	n/a	
Partner/Spouse		9
Other Family member		1

Five key themes from our respondents are highlighted in the section that follows and summarized in Table 2.

### Theme 1: use images and language consistent with how PLWD perceive themselves

Early drafts of the brochure included a vignette highlighting a sample conversation between a patient and her primary care physician. The vignette was accompanied by a photo of a white woman who appeared to be in her upper 70s, wearing tinted glasses and a polka dot dress, with short gray hair, white pearls, and red lipstick on her pursed lips. Participants had a strong reaction to this image. They felt that the image reinforced stereotypes of dementia patients as being elderly, perceived as old-fashioned or past their prime. As one participant put it (PLWD, female): “I see the stigma. I see what everyone thinks of when they hear the word dementia. And I don’t see any of us on this screen that look like that.” Another participant added that upon seeing that image, “Anyone below a certain age doesn’t even open it [the brochure] up” (PLWD, male).

In both the second and third focus group, there was consensus that there should either be no images of patients, or a diverse set of images, so that viewers from various backgrounds might find someone to identify with, regardless of their race, age, or gender. A participant in FG 2 (PLWD, female) said, “If we’re going to use any pictures, let’s represent all of us young people instead of just the stigma of what people think of when they see dementia or hear dementia.” Another participant (CP, female) added, “[….] On commercials on TV sometimes, you’ll see all different ages and ethnicities and that kind of gets the point across, but you’re limited in space here— and somebody would say, oh you left my group out… But you don’t really need three pictures.”

Relatedly, in a section of the brochure labeled “*Did you know?”—*which offered some facts about the escalating risks of overprescribing over time as individuals age—the initial draft drew a distinction between “younger” and “older” adults. The focus group participants included some PLWD in their 50s who did not identify as “older” adults and found this language stigmatizing; they suggested modifying it to be more inclusive. One participant (PLWD, female) said, “We could just say, some people don’t have a problem with the combination of certain drugs, where others do. I think the younger/older part got me.” Rather than singling out older adults, the group instead recommended describing how age can affect the efficacy of certain medications “over time.”

### Theme 2: use straightforward delivery with simple language, activities, and design choices

Early versions of the brochure included a brief quiz with true/false items. This element was modeled after the *EMPOWER* brochure and its approach based on constructivist learning theory [18], which sought to introduce cognitive dissonance related to recipients’ current prescription medication regimens. However, focus group participants expressed strong negative reactions to this mode of presenting information—for PLWD, the quiz felt like an opportunity to fail. Some said it conjured memories of a pop quiz being delivered in school. Then, in addition to the quiz itself causing anxiety, it was constructed such that the correct response for some items was true, while others were false—which also made interpreting the correct answers confusing. One respondent (CP, male) said:

It seems to me that all the answers should either be all true or all false. Because I could see [my wife, who is a PLWD] or my mother coming to me and saying, ‘I got this brochure,’ and I had to kind of think through which wasn’t true, when the other two were. I think I would just make them all true or all false.

As this participant stated, PLWD may find it difficult to keep track of which responses were true and which were false, and presenting facts as questions could impose unnecessary stress.

In addition to the content being a potential source of confusion, the presentation and layout were, as well. A participant in the second focus group (PLWD, female) reported:

“There’s shapes, and there’s black boxes, and there’s different fonts—there’s bold and there’s different sizes of text. […] When I see this, I almost can’t see anything […]. It’s kind of like, when I’m searching for a can of soup at the market—I don’t do that anymore because I just can’t see… I can’t find it. It’s too overwhelming. […] All of a sudden, I can’t…my brain…my eyeballs cannot find what you want me to find.”

For PLWD, not just the content but also the presentation and layout of the content could contribute to information overload.

### Theme 3: avoid content that might heighten fear or anxiety

Participants had strong negative reactions to words or images that conjured fear. The initial draft of the brochure had “You may be at risk” in large letters across the opening panel. While some participants found this message to be an appropriate, engaging opener, the majority were turned off by it. As one participant (PLWD, female) explained:

To me, when I first got it in-in the mail, that ‘You may be at risk’ was a little alarming. […] Every morning I take 8 medicines, and I’m just gobbling all the time. And to me, … for someone with dementia, even an early to moderate case, we already feel at risk. […] [G]et rid of the, ‘You may be at risk,’ […] and take away the scary stuff.

In a subsequent focus group, a participant recommended a “non-alarmist approach,” saying that felt “more respectful.” Another PLWD (female) agreed, saying, “[The] one that…where it just starts out with ‘You may be at risk’- having anxiety, that would set me off. [….] ‘Oh my gosh! I need to get into my doctor right away!’ And then you call your doctor, and you can’t get in for three months. Your anxiety is just going to be off the charts.”

Participants also shared concerns about the image that we initially selected to represent polypharmacy. In the original draft, under the “You may be at risk” text, there was a pile of identical medication capsules with an open pill bottle on its side at the edge of the frame. This was perceived as distressingly off-message for at least one participant, who said she associated that many of the same pills with a suicide attempt (CP, female). Another participant concurred, saying, “It does kind of look like an overdose situation” (CP, male). Instead, the group suggested showing a mix of different pills, which they felt would be a less alarming way to represent their experiences of being prescribed a growing number of medications they were expected to take daily.

### Theme 4: direct recipients to additional information; make the next step easy

Participants expressed an eagerness to find and explore further resources about navigating medications and cognitive impairment, though they expressed uncertainty regarding appropriate next steps. One PLWD (male), a retired healthcare professional explained how a diagnosis of dementia compares to more “typical” or better understood ailments:

If […] diabetes is given to you as a diagnosis, suddenly there is put into motion three or four different parts that the doctor will wind up saying to the nurse or front desk,‘I need Mrs. So-and-So to have the diabetes protocol. I need her to have the information about going to the class about how to give herself injections, how to monitor their diabetes for a sliding scale, send her to a nutrition class…’ But I don’t know how you’d do that with dementia.

He suggested including links to websites in the brochure so patients can find additional information. His care partner agreed, saying, “Send us to some websites where we can educate ourselves a little bit.”

Likewise, a participant in the second focus group (PLWD, male) suggested including links to websites rather than phone numbers: “I mean, our kids would, you know—where can I go on the app? Where can I go online?”

Another participant (CP, female) recommended including the clinic’s phone number directly in the brochure, saying, “While you’ve got it in your hand, it’s a good time to call. I know with [my husband] and his dementia, he does things right away, so he doesn’t forget. So if it’s right in his hand […] or our mind, you can call while it’s in your hand. You probably have the number [somewhere], but let me tell you, if I don’t have to look it up, I like you guys a whole lot more.”

To reiterate, participants described a lack of clarity on next steps once they received a dementia diagnosis, and wanted the brochure to include some concrete, tangible next steps.

### Theme 5: deliver material directly to the PLWD

Participants recommended mailing materials directly to PLWD in a plain, white envelope. Some reported that gimmicky, attention-grabbing junk mail has become so common that they would prefer a simple, straightforward mailing: hand-addressed if possible, with a return address that includes the health system’s name on the front.

“I’m in the position of tossing out a whole bunch of what I call ‘gimme letters,’ be they wonderful organizations or not,” said one care partner (CP, female).

“You don’t open them,” another participant agreed (PLWD, male). One care partner (male) chimed in that a return address from a reputable source is helpful, while another PLWD (male) added, “You want to play up the thing, this is medical, this is [the University]; this is helpful.”

We also asked whether we should address the materials (i.e., the mailing label) to eligible participants, their care partners, or both. Participants were emphatic that the PLWD should be identified and listed first, regardless of their stage of cognitive impairment. One participant (PLWD, female) said, “It does tick me off when I get left out of something that’s meant for me. Then I immediately go to, ‘I know you think I’m stupid, but don’t treat me like a kindergartener.’” Another care partner (female) agreed, saying:

Leaving him off […] would really piss me off, you know. Definitely, out of respect, it should be addressed to the person involved. […] Now, I will probably be the one to open it and read it to [my husband]; he would just, he’d hand it to me. But I definitely think that the person involved should be on it. Spouses or caretakers, that’s okay as a second, sure. Just respect the patient.

**Table 2:** Summary of key findings: Creating written materials for PLWD

Avoid reinforcing stereotypes	Remember that dementia can take many forms and doesn’t only impact the very elderly. Offer materials that represent a diversity of experiences. *“If we’re going to use any pictures, let’s represent all of us young people instead of just the stigma of what people think of when they see dementia or hear dementia.”* (PLWD, female)
Use clean visuals	Avoid unnecessary clutter, changing fonts & text size, overuse of colors. Use high contrast (e.g. navy/white). *“My brain… my eyeballs cannot find what you want me to find.”* (PLWD, female)
Be positive in your approach	A dementia diagnosis can be scary, provoking fear and anxiety. Create materials that help assuage fears, rather than heighten them. *“We already feel at risk. […] Take away the scary stuff.”* (PLWD, female)
Offer concrete action steps	Make it easy for PLWD to take next steps. Include relevant websites and phone numbers where they can get additional information or take a next step. *“Send us to some websites where we can educate ourselves a little bit.”* (CP, female)
Enlist supportive allies—but respect the person	Enlist the support of a care partner—but don’t erase the PLWD; address them too. *“I know you think I’m stupid, but don’t treat me like a kindergartener.”* (PLWD, female)

## Discussion

Through three focus groups, we learned a number of lessons that may be applicable to healthcare researchers and clinicians developing patient-facing education materials for PLWD. Perhaps the most important and overarching lesson is to use an approach that does not unintentionally convey assumptions about PLWD’s identity or autonomy. The assumptions can potentially be avoided in two key ways.

First, those developing materials should carefully consider the pros and cons of using visual representations of individuals chosen to represent the intended target audience. These representations may either not, in fact, reflect the actual participants, or the representations may conflict with how participants perceive themselves. As a result, participants may gain a negative first impression of the material that then makes them unlikely to engage with the written content (e.g., “Anyone below a certain age doesn’t even open it up”).

Secondly, both PLWD and care partners were clear that the material should be addressed to the person with cognitive impairment, even if that person might then ask their care partner to lead the decision-making. Ultimately, the focus groups clearly emphasized the importance of the PLWD remaining the explicit target of the information, and in a way that did not reflect societal stereotypes of what an aging person might look like. In addition to substantive feedback on formatting and content choices that would make written materials easier to engage with, our focus group participants also offered additional feedback regarding the tone of materials specifically designed for PLWD. While their initial reactions to materials designed to induce cognitive dissonance were telling, more research is needed to explore the balance between avoiding undue discomfort to a population that already feels vulnerable, while also provoking sufficient concern to motivate a potential behavioral change.

One of the most successful deprescribing interventions—and the basis of our adapted intervention—was EMPOWER, which used constructivist learning theory intended to affect change by introducing cognitive dissonance related to medication use. However, participants in our focus group described this approach as anxiety-inducing and “scary.” Generally, researchers or clinicians do not want to present information deemed scary, yet some degree of dissonance may be necessary to contribute to behavior change. Future work may be needed to understand whether and how theories of behavior change can apply to PLWD. Finally, separate from the PLWD response to the actual content, the visual presentation of the information may also present challenges in a population that is experiencing change in their visuospatial abilities.

This work does have some limitations. We did not gather information about the type, duration, or severity of the dementia, and participants were those who were interested in engaging in a focus group about these educational materials; therefore, our findings may not be generalizable to all dementia severities or subtypes. In addition, PLWD needed to have a care partner willing to participate (a close friend, romantic partner, or family member), and to be willing and able to join a remote, video call. Therefore, those PLWD with more severe disease or more limited social support or access to technology were under-represented. Furthermore, while our small sample size was appropriate for a qualitative exploration, we acknowledge that perspectives may vary by sociodemographic characteristics such as race, ethnicity, income and level of education, which represent an important area for future dementia research [30]. There was not always consensus among participants; sometimes solving one design problem creates a new one. While we tried to work from a majority model, the nature of a focus group may reward those people more willing to speak publicly and underrepresent the concerns of those who are more reticent to speak. However, the facilitator made efforts to compensate for this by soliciting additional input from quieter participants, explicitly seeking out points of agreement, disagreement, or clarification, and allowing pauses between speakers, rather than immediately launching into the next question. Finally, we offered to provide a final version of the brochure to focus group participants upon request; however, we did not provide a formal process for participants to continue providing additional feedback.

Overall, the feedback from our focus group participants was consistent with best practices recommended by existing scholars of dementia [27, 28]: Materials should be concise and avoid jargon. The content should be relevant and accessible. The style and format should be clean and straightforward. Ample white space should be present, with simple, easy-to-read (sans-serif) fonts. One contribution from our audience was the importance of diversifying the face of dementia, with the understanding that dementia does not only befall those stereotyped patients we may conjure in our minds. In fact, while other scholars recommend use of images, and in particular photographs, our respondents said they’d prefer to have no photos rather than photos that fail to capture a diversity of experiences, thereby alienating their potential audience. Best practices also recommend avoiding images liable to misinterpretation—we learned this firsthand with our image of many pills, which some participants said they associated with suicide risk or overdose. This specific feedback would have been impossible to anticipate without speaking to PLWD themselves.

As the Alzheimer’s Society notes in their Tips for Dementia Friendly Documents [28], however, there is not a single format can meet everyone’s needs. Therefore, they advise getting feedback on people’s experiences with one particular document. The focus groups at the center of this study were an effort to do that. Ultimately, PLWD participating in our focus groups expressed enthusiasm for the opportunity to provide feedback. They welcomed the opportunity to contribute insights that may help researchers and clinicians who are developing PLWD-facing materials. In the end, they wanted to feel seen, heard and included. As the Alzheimer’s Society concludes: “Asking [PLWD] early on about how they like to have information and whether your documents are working for them, can be a great way to give them confidence to speak up and to show you are interested in making change to improve their experience where you can.”

## Conclusion

PLWD-facing educational material should be addressed directly to PLWD, using plain, accessible language and clean, straightforward formatting. Approaches focused on deprescribing that introduce fear or anxiety were not welcome because many PLWD already perceive that they have a high burden of pharmacotherapy and already feel at risk.The study findings may hold relevance for other health-related educational materials aimed at PLWD, providing some inclusive design principles for individuals who may otherwise be excluded and treated inequitably within healthcare services. It will be important going forward to explore ways to create cognitive dissonance that will foster positive change in a population that is prone to anxiety about capabilities and can be easily overwhelmed with information. Furthermore, educational materials will likely need to be tailored to the dementia types and severity, as the type and degree of cognitive impairment will call for different approaches.

### Electronic supplementary material

Below is the link to the electronic supplementary material.

Supplementary Material 1

Supplementary Material 2

## Data Availability

The datasets used and/or analyzed during the current study are available from the corresponding author on reasonable request.

## Abbreviations

AD: Alzheimer’s disease
ADRD: Alzheimer’s disease and Related Dementias
CNS: Central nervous system-active
COREQ: Consolidated Criteria for Reporting Qualitative Research
CP: Care partner
DEEP: Dementia Engagement and Empowerment Project
FG: Focus Group
IMPACT: IMbedded Pragmatic Alzheimer’s disease and AD-Related Dementias Clinical Trials
HIPAA: Health Insurance Portability and Accountability Act
MADRC: Michigan Alzheimer’s Disease Research Center
NCDM: National Council for Dementia Minds
PLWD: Persons living with dementia
