# Supplementary material for: Insights into designing educational materials for persons living with dementia: a focus group study
Source: BMC Geriatr. 2024 Apr 29;24:380. doi: 10.1186/s12877-024-04953-y (PMC11059633; doi:10.1186/s12877-024-04953-y)
Supplement: Supplementary file 1 — Supplementary Material 1 [file 12877_2024_4953_MOESM1_ESM.pdf]

Appendix 1. Focus Group Interview Guide

1. What first catches your eye about the brochure?

2. What, if anything, in the brochure resonates with your experience, as a PLWD or caregiver?

3. What don't you like about the brochure, or what would you change? Why?

4. What would encourage/prompt you to speak with your doctor about your medications? Does the content of this brochure make you feel more or less willing to approach your doctor about making possible changes to medications?

5. What do you think about the length of the brochure? Is there anything you think we should add or remove?

6. Imagine we updated the brochure based on all our feedback today. If you received the brochure in the mail, what would encourage you to actually read the brochure rather than toss it in the recycling bin?

We are planning to mail these to people with mild cognitive impairment or dementia. We are curious in your household, if you received something from your doctor, would you open it yourself? Does somebody else manage your mail? For example, we've heard some people say, "At my parents' house, it doesn't matter who something is addressed to, Mom opens everything."

7. If we were to mail these brochures to PLWDs' homes, what would be the best way to address them? Should they be addressed to the PLWD? A care partner or family member? Both? Someone else?

8. For the PLWD, can you describe the process of making decisions about medications you take such as starting and stopping them? Who all is involved? Is this something you and your care partner discuss? Do you talk about it with your doctor? Anyone else?

9. We are sending the brochure to patients in our clinics as a part of a research study. If you were a participant in this phase of the research study and a recipient of the educational brochure, would you want any follow up information after the study is over? What kind of information would you be interested in receiving?

10. Is there anything else you wanted to share with us today that you have not had a chance to?
