# Supplementary material for: Insights into designing educational materials for persons living with dementia: a focus group study
Source: BMC Geriatr. 2024 Apr 29;24:380. doi: 10.1186/s12877-024-04953-y (PMC11059633; doi:10.1186/s12877-024-04953-y)
Supplement: Supplementary file 2 — Supplementary Material 2 [file 12877_2024_4953_MOESM2_ESM.pdf]

Appendix 2. Tri-Fold Brochure- Front/outside & Inside

Questions for your Doctor or Pharmacist

1. Could we review why I am taking these medications?
2. Do I need to take these medications forever?
3. Is there at least one medication that I could safely reduce or stop?
4. If we decide to reduce or stop one medication, what symptoms should I look out for?

Use this space to write other questions you have about your medications:

Next Steps

- Bring this brochure and a list of questions to your next doctor's appointment.
- If you don't have your next appointment scheduled, make one!

Talk to your doctor first before making any medication changes.

Resources

- **Your local Area Agency on Aging**
  aaa1b.org
- **The Alzheimer's Association**
  alz.org
- **The National Council for Dementia Minds**
  dementiaminds.org
- **Positive Approach to Care**
  teepasnow.com
- **Health in Aging**
  healthinaging.org/medications-older-adults

Is it time to check with your doctor about the medications you take?

Too many medications may do more harm than good.

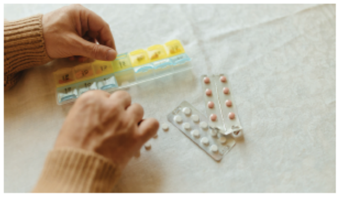

MICHIGAN MEDICINE
UNIVERSITY OF MICHIGAN

HENRY FORD HEALTH

Did you know?

Some medications for pain, sleep, mood, or anxiety—especially when taken in combination—may negatively affect your brain and thinking.

New research shows that taking these kinds of medication combinations may leave your thinking fuzzy or your memory worse.

As you age, your body processes medication differently. Over time, some combinations may increase fatigue and your risk of falling.

You may be able to reduce or even stop one or more medications. Taking fewer medications may help you feel better and experience fewer side effects.

Medications that are commonly part of these combinations:

- Cymbalta (duloxetine)
- Desyrel (trazodone)
- Neurontin (gabapentin)
- Remeron (mirtazapine)
- Wellbutrin (bupropion)

The full list of medications can be found here:

Frequently asked questions

1. I have taken my medications for many years. Is it ok to keep taking them as I age?

Medications may affect your body differently as you age. Your body also processes or breaks down medication differently. This means that a medication you have been taking for a long time could be less safe than when you first started it.

2. How do I know if a medication is causing side effects?

This is not always easy to tell, especially if you have been taking a medication for a long time. Also, people may think symptoms like memory troubles or feeling unsteady on their feet are just part of getting older. But in fact, these and other symptoms could be medication side effects.

3. I don't think I am having any side effects. Why should I stop a medication?

Any medication can have side effects. Whether you should stop one depends on what you're taking & why. Some medications like opioids & benzodiazepines are intended for short-term use. That's why it's good to review what you're taking with your doctor.
